# Heterotopic Ossification: An Unusual Presentation

**DOI:** 10.1155/2012/516717

**Published:** 2012-12-31

**Authors:** Satish G. Patil, Aaisha Siddiqua, Udupi Krishna Joshi, Pallavi K. Deshmukh, Bindu S. Patil, Anand Mangalgi

**Affiliations:** ^1^Department of Oral and Maxillofacial Surgery, S. Nijalingappa Institute of Dental Sciences and Research, Gulbarga, Karnataka 585105, India; ^2^Department of Oral Medicine and Radiology, S. Nijalingappa Institute of Dental Sciences and Research, Gulbarga, Karnataka 585105, India; ^3^Department of Periodontics, S. Nijalingappa Institute of Dental Sciences and Research, Gulbarga, Karnataka 585105, India

## Abstract

Heterotopic ossification (HO) is usually seen after-trauma, following traumatic injuries, surgeries involving major joints, neurogenic injury, and burns; however, atraumatic cases have also been reported. HO tends to cause pain, swelling, and limitation of joint movements. HO has been reported in adults as well as in pediatric cases, however, our search in the English literature has not revealed a single case in the infratemporal region, especially in children of developing age, where HO tends to affect the development and growth of adjacent bones. We are reporting a case of HO in close proximity to TMJ affecting the development of mandible and maxilla.

## 1. Introduction

Heterotopic ossification is the formation of mature lamellar bone in soft tissues outside the joint capsule and periosteum. HO usually occurs secondary to trauma and has been frequently reported after hip arthroplasty and spinal injury. It is usually symptomatic, and patient presents with pain and swelling. Cases showing limited range of motion, due to presence of HO in close proximity to joint, have been reported. However, literature has not revealed HO affecting the TMJ and its development. A case of one such patient, where HO occurred in close proximity to TMJ affecting its function and growth of maxilla and mandible, is reported. The incomplete development of maxilla and mandible resembles a mild case of Goldenhar syndrome. Until further evidence disproves this, this may be considered first such case of Goldenhar syndrome with HO.

## 2. Case Report

**Figure 1:**
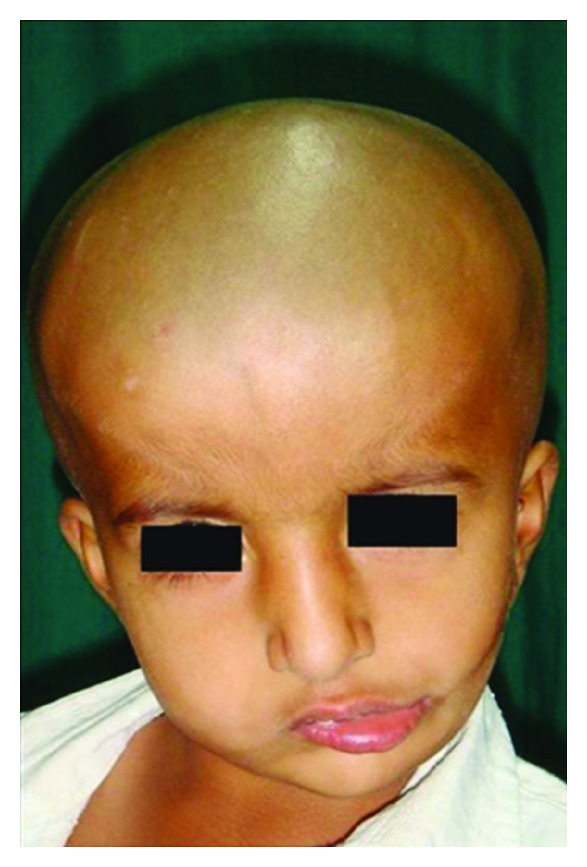
Preoperative frontal view.

A four-year-old female child patient reported to the Department of Oral Surgery with her parents who complained of restricted mouth opening and asymmetry of face. History revealed that, the child was born with an apparently normal face and adequate mouth opening. Deviation of mouth and gradual reduction in mouth opening were noticed from the second year of life. Clinical examination revealed an obvious asymmetry of lower third of face (Figure 1). There was fullness of face on the right side as compared to the left side with a shallow groove in the left cheek at the junction of middle and lower one-third of the face, extending from the left angle of mandible and disappearing over the ramus. On opening of mouth, an obvious deviation of chin towards left side was noted. Preauricular skin tags were noticed on both sides, with the left one being more prominent.

**Figure 2:**
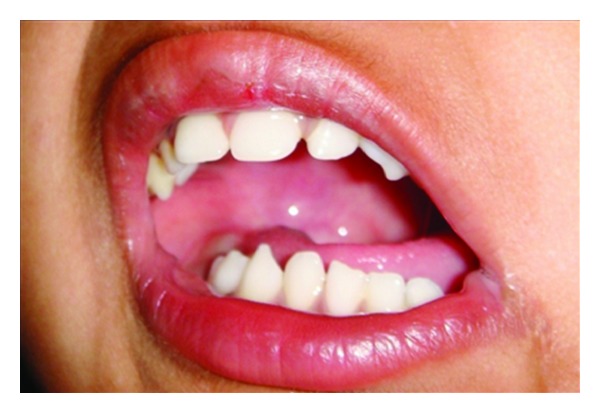
Deviated and restricted mouth opening.

**Figure 3:**
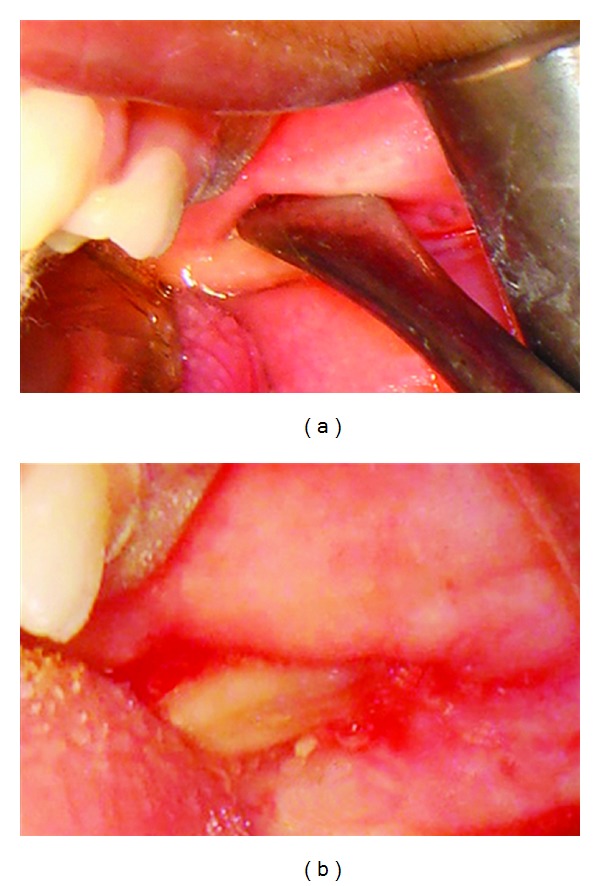
(a) Intraoral photograph showing the mass, (b) irritational Fibroma.

Intraoral examination revealed a shift in midline towards left with the upper midline coinciding with the lower right canine with an overjet of 15 mm. Mouth opening was restricted to 2 cm (Figure 2). On palpation, a bony hard mass was felt in the posterosuperior part of the upper left buccal vestibule below the mucosa (Figures 3(a) and 3(b)). Boundaries could not be confirmed. The mass seemed to extend into the infratemporal region. Dimpling of the mucosa was present right below the mass and 1 cm × 1 cm of pale, and firm tissue could be palpated. History of trauma was not precise and could not be confirmed. There was no history of pain. 

**Figure 4:**
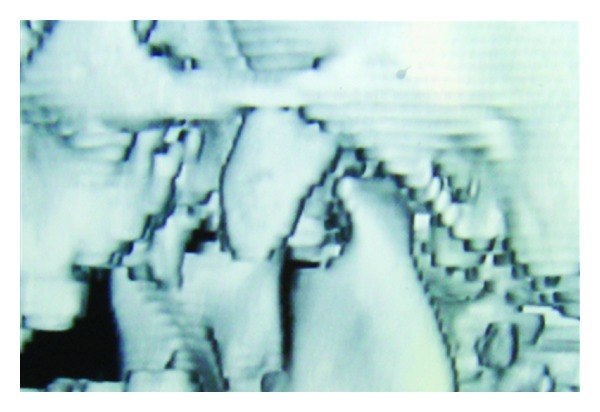
Computed tomography-lateral view.

**Figure 5:**
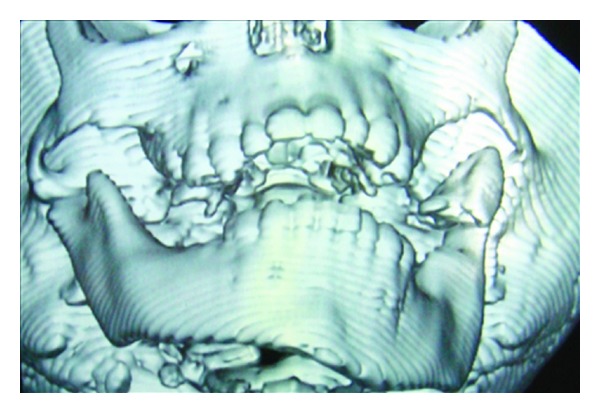
Computed tomography-frontal view.

Computed tomography revealed a bony structure measuring 3 cms by 1.5 cms, roughly conical in shape in the infratemporal region, in front of the condyle and was overlying the coronoid process (Figures 4 and 5). The apex extended medial to the zygomatic arch. The bony mass was not attached to any of the adjacent. An abnormal development of the maxilla and mandible with a shift of the midline of the mandible to the left was seen. The anteroposterior growth of the right side of the mandible was more than that of the left side. The left coronoid was small representing stunted growth. The condyle seemed to be normal in structure. Based on the clinical and radiological findings a provisional diagnosis of heterotopic ossification was made. 

**Figure 6:**
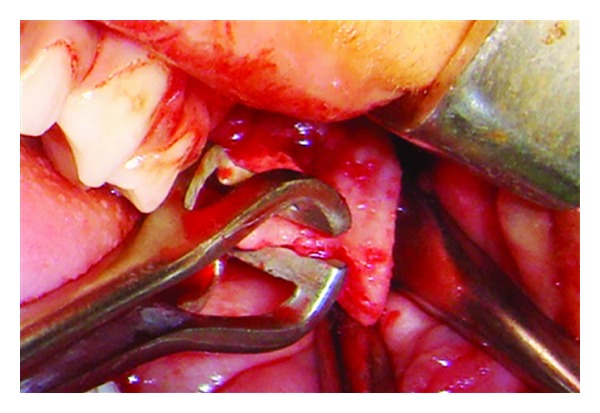
Intraoperative photograph showing excision of the lesion.

**Figure 7:**
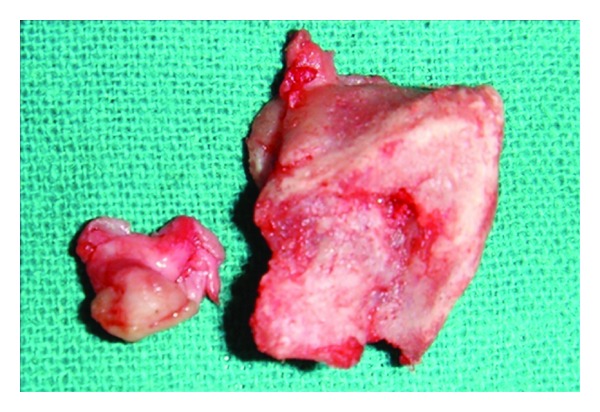
Excised osseous mass and soft tissue.

**Figure 8:**
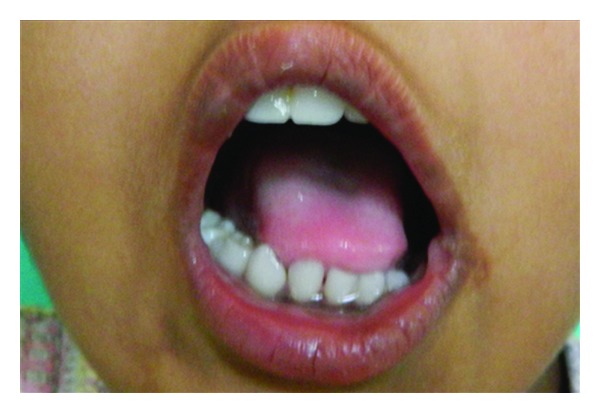
Adequate mouth opening postoperatively.

Surgical procedure for excision of the bony mass was performed under general anesthesia. The bony mass was palpated, and a vertical mucosal incision was given over it. Blunt dissection was performed to expose the bony mass (Figure 6). The bony mass was easily separated from the surrounding tissues and removed in toto (Figure 7). The fibrous tissue adjacent to the mass was excised. Hemostasis was achieved, and site was closed with 3–0 vicryl. Postoperative period was uneventful. 3 years postoperatively patient showed adequate mouth opening (Figure 8). Histopathologic examination of the mass revealed presence of mature lamellar bone.

## 3. Discussion 

Heterotopic ossification is the formation of mature lamellar bone in soft tissues. It forms outside the joint capsule and periosteum. It is usually symptomatic but can remain asymptomatic. When symptomatic, it causes pain, swelling and can present with functional impairment which is seen as limited range of motion when in proximity to a joint [1, 2]. Symptoms depend on the size and anatomical site.

The most common cause of HO is trauma [2], although, atraumatic HO has also been reported. Trauma could be in the form of musculoskeletal injury, surgical trauma, or warfare injuries. Other causes of HO are hereditary [3], burns [4], and neurogenic injury [2]. HO can occur in muscle, adipose tissue, and connective tissue.

Pathogenesis: although a precise pathophysiology of HO is yet to be made clear, a lot of studies indicate multifactorial process following trauma. Studies indicate role of various local and humoral factors [2, 5]. The role of BMP, osteogenic precursor cells, an appropriate environment that results following injury, is necessary for HO, with BMP induction causing the differentiation of precursor cells to osteoblasts. The precursor cells could be dormant osteoprogenitor cells [4], mesenchymal cells which are locally present or have migrated to the site of injury [5], or cells of vascular origin [2]. In neurogenic HO, studies suggest that BMP induction results in migration/release of osteogenic and other stem cells from the nerve [6]. 

 Very few cases of HO have been previously reported in the literature in the maxillofacial region. Myositis ossificans has been reported in muscles of mastication [7]. A case of atraumatic HO in the scalp [8], HO following transposition of temporalis muscle in the cheek for the treatment of facial paralysis [9] and panniculitis ossificans in submental region [4], has been reported.

Our case is unique as she presents with HO at a very young age, at a very unusual site, and in connective tissue. As the child was in the growing age, the growth of both maxilla and mandible was affected on the involved side by the presence of bony mass. The mandible showed reduced anteroposterior growth of the body of the mandible and stunted growth of the coronoid on the affected side, resulting in asymmetrical mandible. Mild canting of the maxillary occlusal plane indicating reduced maxillary growth was noted. 

In view of the presence of preauricular skin tags, first arch syndrome may be implicated. The features are consistent with the findings of Goldenhar syndrome. Goldenhar syndrome is a developmental anomaly of maxillofacial skeleton and hemifacial soft tissue. Characteristic features include facial asymmetry, hemifacial microsomia, microphthalmia, mandibular hypoplasia, unilateral ear deformity, and preauricular tags or sinuses. The internal organs of the central nervous system, cardiovascular system, renal system, respiratory system, and the gastrointestinal system may also be underdeveloped or sometimes absent [10]. Our patient presents with an asymmetrical face with deviation of lower third of face to the left indicating deficient growth of the jaws on left side. No previous case of HO in Goldenhar syndrome or any developmental syndrome has been previously reported. This case can be considered a case of Goldenhar syndrome with mild representation and the first reported case of HO in Goldenhar syndrome. 

Based on the clinical and radiological findings a differential diagnosis should include myositis ossificans circumscripta, myositis ossificans progressive, osteoma, nodular fasciitis, osteosarcoma, and chondrosarcoma. Slowly calcifying lesions synovial sarcoma, rhabdomyosarcoma, and malignant fibrous histiocytoma should also be included [7]. The most important pathology to be excluded is osteosarcoma. Histopathologic finding of mature lamellar bone confirms the diagnosis of HO.

Postsurgically HO has been found to occur most frequently following total hip arthroplasty, hence various preventive modalities of HO have been discussed in the literature for such cases. Different modalities used include diphosphonates and NSAID's such as indomethacin and naproxen. Radiotherapy has also been used to reduce the incidence of HO in orthopedic management. Noggin, a BMP inhibitor, pulsed electromagnetic fields (PEMF), and free radical scavengers in the form of allopurinol and N-acetylcysteine are the three new methods being evaluated [11].

Patients present with various complaints as previously discussed. If radiographic examination and bone scanning reveal HO in formative stage, it is recommended to provide symptomatic treatment and wait for complete maturation of bone. Surgical excision is done thereafter. It has also been reported to either regress or stabilize. 

## 4. Conclusion

HO can occur in pediatric age group in the maxillofacial region and affect the development and growth of the TMJ. History of trauma and symptoms like pain, swelling, and limited range of motion related to HO may or may not be present. When patient presents with facial asymmetry, restricted mouth opening, and improper development of jaws, HO and similar lesions like osteoma should be included in the provisional diagnosis. Early diagnosis and management can prevent the developmental abnormalities, which result in loss of function and esthetics.

## References

[1] Onder K, Muhammed B, Saime U, Haluk A (2011). Post-traumatic heterotopic ossification of the crus: a case study. *Ortopedia Traumatologia Rehabilitacja*.

[2] Hsu JE, Keenan MA (2010). Current review of heterotopic ossification. *Journal of Orthopaedics*.

[3] Shore EM, Kaplan FS (2010). Inherited human diseases of heterotopic bone formation. *Nature Reviews Rheumatology*.

[4] Burke GAE, Shah D, MacBean AD (2008). Panniculitis ossificans traumatica: an unusual presentation. *British Journal of Oral and Maxillofacial Surgery*.

[5] Yildiz N, Ardiç F (2010). Pathophysiology and etiology of neurogenic heterotopic ossification. *Turkish Journal of Physical Medicine and Rehabilitation*.

[6] Salisbury E, Rodenberg E, Sonnet C (2011). Sensory nerve induced inflammation contributes to heterotopic ossification. *Journal of Cellular Biochemistry*.

[7] Saussez S, Blaivie C, Lemort M, Chantrain G (2006). Non-traumatic myositis ossificans in the paraspinal muscles. *European Archives of Oto-Rhino-Laryngology*.

[8] Müller CSL, Rass K, Tilgen W (2010). Panniculitis ossificans non-traumatica of the scalp. *Journal of Cutaneous Pathology*.

[9] Adler N, Yaffe B (2005). Ectopic bone formation following temporalis muscle transposition for facial paralysis: a rare complication?. *Annals of Plastic Surgery*.

[10] Castriota-Scanderbeg A, Dallapiccola B, Heilman U (2005). *Abnormal Skeletal Phenotypes: from Simple Signs to Complex Diagnosis*.

[11] Baird EO, Kang QK (2009). Prophylaxis of heterotopic ossification-an updated review. *Journal of Orthopaedic Surgery and Research*.

